# Value of ^68^Ga-labeled bombesin antagonist (RM2) in the detection of primary prostate cancer comparing with [^18^F]fluoromethylcholine PET-CT and multiparametric MRI—a phase I/II study

**DOI:** 10.1007/s00330-022-08982-2

**Published:** 2022-07-21

**Authors:** Mohsen Beheshti, Pekka Taimen, Jukka Kemppainen, Ivan Jambor, Andre Müller, Wolfgang Loidl, Esa Kähkönen, Meeri Käkelä, Mathias Berndt, Andrew W. Stephens, Heikki Minn, Werner Langsteger

**Affiliations:** 1 Division of Molecular Imaging and Theranostics, Department of Nuclear Medicine & Endocrinology, University Hospital Salzburg, Paracelsus Medical University, Muellner Hauptstrasse 48, A-5020 Salzburg, Austria; 2 Department of Nuclear Medicine & Endocrinology, PET-CT Center LINZ, St. Vincent’s Hospital, Linz, Austria; 3 Institute of Biomedicine, University of Turku, Finland and Department of Pathology, Turku University Hospital, Turku, Finland; 4 Turku PET Center, Department of Nuclear Medicine, University of Turku, Turku, Finland; 5 Department of Radiology, Icahn School of Medicine at Mount Sinai, New York, USA; 6 Department of Radiology, University of Turku, Turku, Finland; 7 Medical Imaging Centre of Southwest Finland, Turku University Hospital, Turku, Finland; 8 Life Molecular Imaging GmbH, Berlin, Germany; 9 Department of Urology, Ordensklinikum, Linz, Austria; 10 Department of Urology, Turku University Hospital, Turku, Finland; 11 Institute of Clinical Medicine, Department of Clinical Oncology, Turku University Hospital, Turku, Finland; 12 Department of Nuclear Medicine, Medical University of Vienna, Vienna, Austria

**Keywords:** Prostate cancer, [^68^Ga]Ga-RM2, [^18^F]FCH, PET-CT, mpMRI

## Abstract

**Objectives:**

The bombesin derivative RM2 is a GRPr antagonist with strong binding affinity to prostate cancer (PCa). In this study, the impact of [^68^Ga]Ga-RM2 positron emission tomography-computed tomography (PET-CT) for the detection of primary PCa was compared with that of [^18^F]FCH PET-CT and multiparametric magnetic resonance imaging (mpMRI).

**Methods:**

This phase I/II study was conducted in 30 biopsy-positive PCa subjects. The patients were stratified into high (10 patients), intermediate (10 patients), and low risk (10 patients) for extraglandular metastases as defined by National Comprehensive Cancer Network (NCCN) criteria (NCCN Clinical Practice Guidelines in Oncology, 2016). The prostate gland was classified in 12 anatomic segments for data analysis of the imaging modalities as well as histopathologic findings. The segment with the highest radiotracer uptake was defined as the “index lesion.” All cases were scheduled to undergo prostatectomy with pelvic lymph node (LN) dissection in intermediate- and high-risk patients. Intraprostatic and pelvic nodal [^68^Ga]Ga-RM2 and [^18^F]FCH PET-CT findings were correlated with mpMRI and histopathologic results.

**Results:**

Of the 312 analyzed regions, 120 regions (4 to 8 lesions per patient) showed abnormal findings in the prostate gland. In a region-based analysis, overall sensitivity and specificity of [^68^Ga]Ga-RM2 PET-CT in the detection of primary tumor were 74% and 90%, respectively, while it was 60% and 80% for [^18^F]FCH PET-CT and 72% and 89% for mpMRI. Although the overall sensitivity of [^68^Ga]Ga-RM2 PET-CT was higher compared to that of [^18^F]FCH PET-CT and mpMRI, the statistical analysis showed only significant difference between [^68^Ga]Ga-RM2 PET-CT and [^18^F]FCH PET-CT in the intermediate-risk group (*p* = 0.01) and [^68^Ga]Ga-RM2 PET-CT and mpMRT in the high-risk group (*p* = 0.03). In the lesion-based analysis, there was no significant difference between SUVmax of [^68^Ga]Ga-RM2 and [^18^F]FCH PET-CT in the intraprostatic malignant lesions ([^68^Ga]Ga-RM2: mean SUVmax: 5.98 ± 4.13, median: 4.75; [^18^F]FCH: mean SUVmax: 6.08 ± 2.74, median: 5.5; *p* = 0.13).

**Conclusions:**

[^68^Ga]Ga-RM2 showed promising PET tracer for the detection of intraprostatic PCa in a cohort of patients with different risk stratifications. However, significant differences were only found between [^68^Ga]Ga-RM2 PET-CT and [^18^F]FCH PET-CT in the intermediate-risk group and [^68^Ga]Ga-RM2 PET-CT and mpMRT in the high-risk group. In addition, GRP-R-based imaging seems to play a complementary role to choline-based imaging for full characterization of PCa extent and biopsy guidance in low- and intermediate-metastatic-risk PCa patients and has the potential to discriminate them from those at higher risks.

**Key Points:**

• [^*68*^*Ga*]*Ga-RM2 is a promising PET tracer with a high detection rate for intraprostatic PCa especially in intermediate-risk prostate cancer patients.*

• *GRPr-based imaging seems to play a complementary role to choline-based or PSMA-based PET/CT imaging in selected low- and intermediate-risk PCa patients for better characterization and eventually biopsy guidance of prostate cancer disease.*

**Supplementary Information:**

The online version contains supplementary material available at 10.1007/s00330-022-08982-2.

## Introduction

Given the multifocal nature of prostate cancer, the accurate imaging and determination of its extent make it one of the most challenging malignancies. Prostate-specific antigen (PSA) and digital rectal examination followed by transrectal ultrasound (TRUS)–guided biopsies are the standard approaches in the primary assessment of prostate cancer [1]. Due to the low diagnostic accuracy of TRUS, systematic biopsies are frequently used for the detection of prostate cancer [1]. Nevertheless, approximately one-third of cancers are missed on initial systematic biopsies [2, 3] and the Gleason score is upgraded between biopsies and radical prostatectomy [4]. Additionally, attempts to improve prostate cancer detection by intensifying the biopsy technique have not proven successful and appear to cause an increase in the risk of complications [5]. Therefore, there is an increasing need for accurate imaging modalities to guide biopsy and avoid related complications.

Multiparametric magnet resonance imaging (mpMRI) has been shown to improve diagnostic accuracy in the evaluation of intraglandular prostate cancer [6]. However, high inter-reader and inter-center variability has been reported, limiting the widespread use of mpMRI in men with diagnosed and/or suspected prostate cancer [6, 7].

Cancer diagnosis draws on the use of molecular imaging as one of its essential tools. Currently, positron emission tomography (PET) and computed tomography (CT) play a pivotal role among molecular imaging modalities, providing noninvasive information which is functional as well as anatomical. In the last decade, several PET radiotracers have been investigated through clinical trials to form an accurate depiction of intraglandular malignancies in the prostate [8–13]. PET/CT using ^11^C- and ^18^F-labeled choline has shown consistent reliability in diagnostic performance in the assessment of recurrent prostate cancer [9, 14]. However, its inability, in the pre-operative setting, to accurately differentiate cancerous tissues from inflammatory lesions or benign prostatic hyperplasia (BPH) should be noted [10].

Gastrin-releasing peptide receptor (GRPr) proteins are highly overexpressed in multiple human tumors and have been detected in 63–100% of human prostate cancer tissue [15, 16]. ^68^Ga-labeled-DOTA-4-amino-1-carboxymethylpiperidine-DPhe-Gln-Trp-Ala-Val-Gly-His-Sta-Leu-NH2 ([^68^Ga]Ga-RM2) is a synthetic bombesin receptor antagonist with high binding affinity to GRPr [17]. [^68^Ga]Ga-RM2 PET has shown significant potential for imaging of primary prostate cancer in previous preclinical studies [18, 19]. Recently, prospective clinical trials have been conducted using [^68^Ga]Ga-RM2 PET-CT both in staging and in recurrent prostate cancer. The primary results were promising [20, 21].

Because [^68^Ga]Ga-RM2 and ^18^F-choline target different biologic processes, understanding how these two tracers behave in PCa patients with different tumor characteristics and risks of metastasis is essential for determining the best management scenario.

The high diagnostic performance of PET/CT imaging using radiolabeled prostate-specific membrane antigen (PSMA) in the assessment of prostate cancer has been shown in a large number of recent published data, particularly in the recurrent setting of the disease. However, the present prospective dual-center clinical trial was designed few years ago, at a time when [^68^Ga]Ga-PSMA was not widely available. This study was conducted to compare the diagnostic potential of PET-CT imaging for the detection of primary prostate cancer with [^68^Ga]Ga-RM2, as a novel PET tracer, and imaging using [^18^F]fluoromethyl-dimethyl-2-hydroxyethylammonium ([^18^F]FCH) and to correlate the results with mpMRI. In addition, we examined the diagnostic performance of these two tracers and mpMRI in low-, intermediate-, and high-risk prostate cancer patients for intraprostatic cancer detection and extraglandular metastases. To our best knowledge, this is the earliest instance of such a study design being conducted with humans.

## Materials and methods

### Patients

This prospective exploratory phase I/II two-center clinical trial was performed in accordance with the principles of the 1964 Declaration of Helsinki and its later amendments or comparable standards and was approved by the ethics committees of the study centers. The study was performed in two centers (Ordensklinikum Linz, Austria, and University of Turku, Finland) and has been also registered in the European Clinical Trial Register with the register number of EudraCT-Nr.: 2014-003027-21. Signatures of the written informed consent were obtained from all subjects of the study.

The study was performed in primary staging of 30 men with biopsy-proven prostate cancer. The patients were stratified into high (10 patients), intermediate (10 patients), and low risk (10 patients) for extraglandular metastases as defined by National Comprehensive Cancer Network (NCCN) criteria [22].

The inclusion and exclusion criteria are shown in supplement 1. Radical prostatectomy was performed in all patients within a maximum interval of 4 weeks after completing the imaging modalities. In 4 patients, an old partial transurethral prostatectomy has been performed which was not pointed out by patients and was not apparent in the primary medical history of these patients. Therefore, they were excluded to avoid any bias in the results of this study. The data from 26 patients (9 low risk, 8 intermediate risk, and 9 high risk) were finally analyzed.

Safety monitoring included physical examination, electrocardiography, and laboratory parameters of various organs performed during the [^68^Ga]Ga-RM2 PET-CT examination and 24 h as well as 3–5 days after radiotracer injection. Adverse events were documented. The common pattern of physiologic [^68^Ga]Ga-RM2 PET biodistribution in different anatomical structures has been documented.

### PET-CT imaging and data analysis

The patients underwent [^68^Ga]Ga-RM2 PET-CT and [^18^F]FCH PET-CT (all except 3 patients) with a maximum interval of 30 days (average: 8.7 days, range: 1–29 days). Imaging modalities have been performed at least 2 weeks after prostate biopsy.

The study was performed with dedicated PET-CT scanners (GE Healthcare, 23 patients: Discovery 710; 3 patients: Discovery VCT). Imaging was acquired 60 min after an intravenous (i.v.) injection of 4 mCi (148 MBq) of ^68^Ga-RM2. All except 3 patients had concomitant ^18^F-FCH PET-CT imaging 60 min after i.v. administration of 6 mCi (222 MBq) [^18^F]FCH.

PET acquisitions were obtained from the base of the skull to the proximal of the thigh with 2.5 min/bed position acquisition time using time-of-flight (TOF) modus. All images were reconstructed identically with ordered-subsets expectation maximization algorithm (4 iterations, 18 subsets) followed by a post-reconstruction smoothing Gaussian filter (4.0 mm in full width at one-half maximum).

The CT portion of the ^18^F-FCH PET-CT procedure was performed after intravenous infusion of 100 ml ionic contrast medium with high beam current modulation (120–330 mA, 0.6 s per rotation, 5.0 mm reconstructed section thickness, 0.5 mm overlap, 512 × 512 matrix, pitch index 1.5), while a non-contrast CT with low beam current modulation (80–120 mA) was obtained on 68Ga-RM2 PET-CT for localization and attenuation correction. The reformatted, transverse, coronal, and sagittal views were used for interpretation.

Images were read using advanced PET-CT review software (Advantage Windows, version 4.6; GE Medical Systems). More details of the PET-CT imaging are described in supplement 2. A lesion was considered pathologic when the focal tracer accumulation was greater than the background activity. The common pattern of physiologic radiotracer distribution of 68Ga-RM2 in the study cohort has been recorded.

### MRI—imaging and data analysis

Each patient was examined in the supine position in 1.5-T (*n* = 23) and 3-T (*n* = 3) scanners (Siemens, Erlangen, Germany) using surface phased-array coils or a combination of surface phased-array coils and endorectal coil.

MR data sets (T2 weighted (T2W), apparent diffusion coefficient (ADC), and dynamic contrast enhanced (DCE)) were evaluated qualitatively based on the PI-RADS (version 2.1) scoring system for the tumor detection and localization. mpMRI images have been reported by experienced radiologists in each study center in the setting of daily work routine. In addition, central (external) reading was performed by an experienced radiologist (> 10 years). Further details of prostate MRI protocol are presented in supplement 2.

### Data reading and final interpretation

**Fig. 1:**
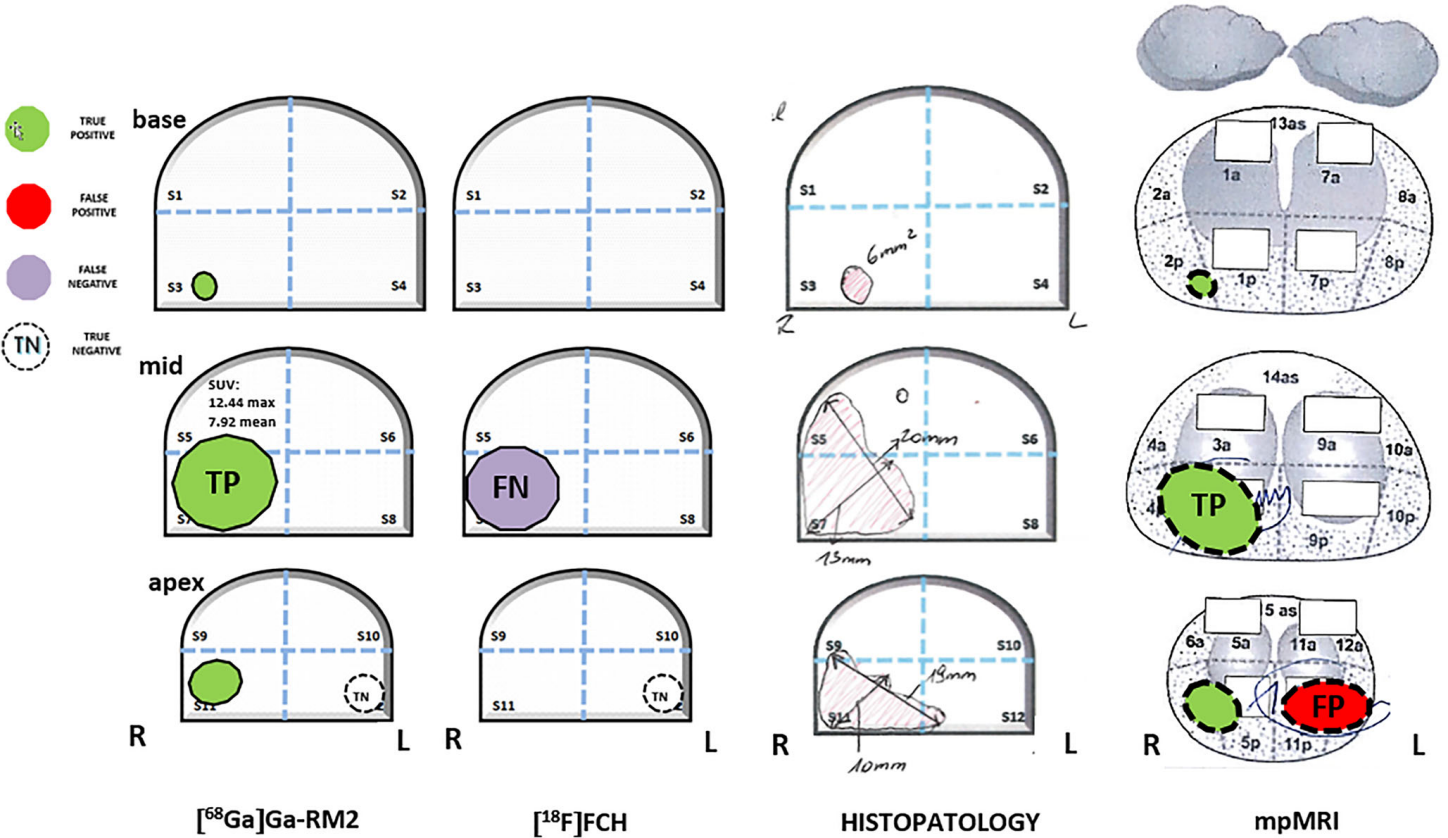
Schematic presentation of the methodological approach for segmentation of the prostate gland and systematic reading of imaging modalities and correlation with histopathological findings. It refers to the data presented on Fig. 2

A systematic approach of double reading was performed for all imaging and histopathologic data (Fig. 1). The prostate gland was classified into 12 anatomic segments for data analysis of both imaging and histopathologic findings. The segment with the highest radiotracer uptake was defined as the “index lesion” on PET/CT.

mpMRI images were read both in the setting of clinical routine as well as central external reading by experienced radiologists (> 10 years).

The final diagnosis of imaging results was based on histopathological findings as “gold standard.” All data and the discrepancies between readers have been discussed in a consensus meeting, in that all experts from different disciplines participated. The final interpretation of the imaging findings was made by reviewing the histopathological results and a consensus between readers.

## Statistical analysis

Univariate analysis was performed to assess the variables and frequency tables. Quantitative variables were summarized using descriptive statistics (mean ± SD) and were compared in different groups using the independent *t* test. The paired *t* test was used to compare quantitative variables in a paired group. Sensitivity and specificity were calculated using data collected from PET studies on a per-region basis. Clopper-Pearson’s correlation coefficient was calculated for correlations between different quantitative variables. Statistical analysis was conducted using SPSS software version 24 (SPSS Inc.). A value of *p* < 0.05 was considered to indicate statistical significance in all comparisons.

## Results

### Biodistribution and safety

No adverse clinical reactions, abnormal laboratory findings, or side effects were detected during the 3–5 days after intravenous administration of [^68^Ga]Ga-RM2.

**Fig. 2:**
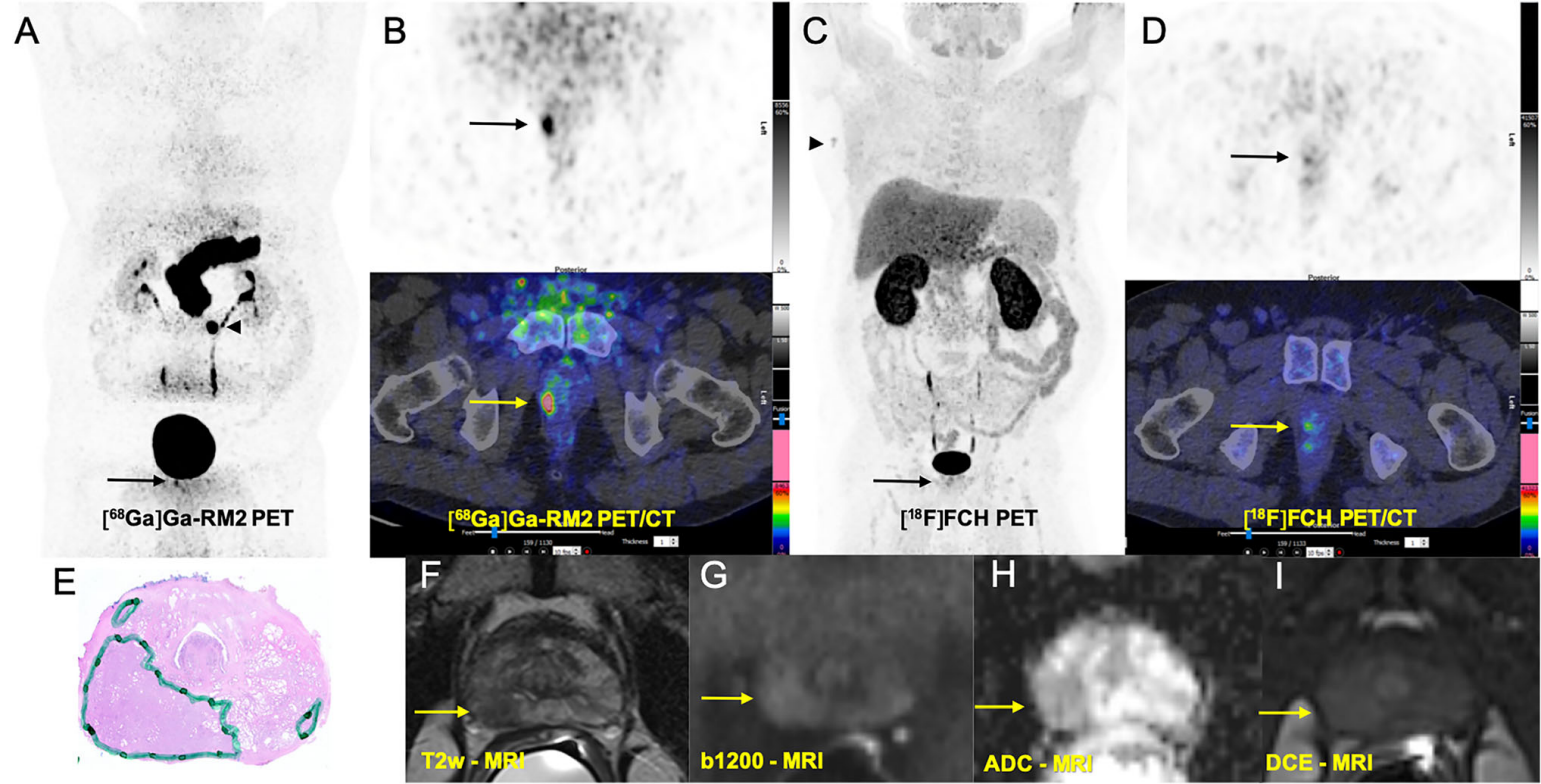
Comparison of [68Ga]Ga-RM2 PET-CT with [18F]FCH PET-CT and mpMRI in pre-operative staging of a prostate cancer patient with intermediate risk of extraglandular metastases (PSA: 9.0 ng/ml, Gleason score: 7 (4 + 3), grade 3, TNM: pT2c N0 (0/14) R0 L0 V0 Pn1). **A** [^68^Ga]Ga-RM2 PET: maximum intensity projection (MIP) shows intensive physiologic tracer uptake in the pancreas with a focal non-specific bowel uptake on the middle of the left abdomen (arrowhead). A focal tracer uptake is evident in the right prostate lobe (arrow). **B** Axial view [^68^Ga]Ga-RM2 PET-CT (PET: upper, fusion PET-CT: middle) from the prostate region showing intensive focal uptake on the left prostate lobe (arrows) corresponding with the findings on histopathology (malignancy is marked) (**E**). [^18^F]FCH PET-CT MIP (**C**) axial view of the prostate region (**D**): mild focal uptake is seen on the right axillary region, suggestive of reactive lymph node. No appreciable [^18^F]FCH uptake is seen on the prostate. T2-weighted image (**F**) demonstrated a focal area of decreased signal in the left peripheral zone (yellow arrow) with corresponding diffusion-weighted imaging signal restriction (trace *b* values of 1200 s/mm^2^, **G**; ADC, **H**) and early contrast enhancement (dynamic contrast enhancement, DCE, **I**). Overall, these findings represented a PI-RADs version 2.1 score 5 lesion

**Fig. 3:**
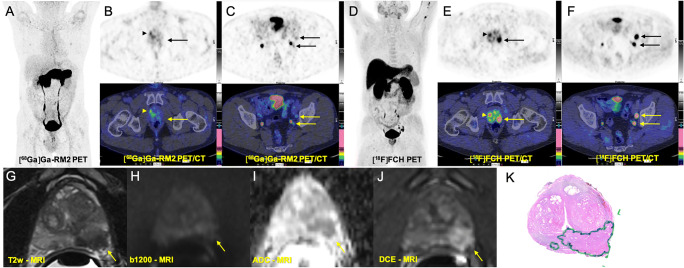
Comparison of [^68^Ga]Ga-RM2 PET-CT with [^18^F]FCH PET-CT and mpMRI in pre-operative staging of a prostate cancer patient with high risk of extraglandular metastases (PSA: 30.0 ng/ml, Gleason score: 8 (4 + 4), grade 3, TNM: pT3b pN1 (3/14) R1 (Apex li) L0 V0 Pn1). **A** [^68^Ga]Ga-RM2 PET: maximum intensity projection (MIP) shows mild focal tracer uptake on the right prostate lobe (arrowhead). **B** Axial view [^68^Ga]Ga-RM2 PET-CT (PET: upper, fusion PET-CT: lower row) from the prostate region shows mild focal tracer uptake on the right prostate lobe (arrowhead) without corresponding malignant findings on histopathology (false positive). **C** Axial view [^68^Ga]Ga-RM2 PET-CT (PET: upper, fusion PET-CT: lower row) from the pelvis shows only faint tracer uptakes on the lymph nodes on the left iliac region (arrows). **D** [^18^F]FCH PET-CT MIP. **E** Axial view [^18^F]FCH PET-CT (PET: upper, fusion PET-CT: middle) from the prostate region shows intensive focal tracer uptake on the left prostate lobe (arrows) corresponding with malignant findings on histopathology **(K)** (true positive). A focal tracer uptake is also seen on the right prostate lobe (arrowhead) without corresponding malignant findings on histopathology (false positive). **F** Axial view [^18^F]FCH PET-CT from the pelvis shows intensive focal tracer uptakes on the lymph nodes on the left iliac region (arrows), verified as metastases on histhopathology (true positive). T2-weighted image (**G**) demonstrated a focal area of decreased signal in the right peripheral zone (yellow arrow) with corresponding diffusion-weighted imaging signal restriction (trace *b* values of 1200 s/mm^2^, **H**; ADC, **I**) and early contrast enhancement (dynamic contrast enhancement, DCE, **J**). Overall, these findings represented a PI-RADs version 2.1 score 5 lesion

**Fig. 4:**
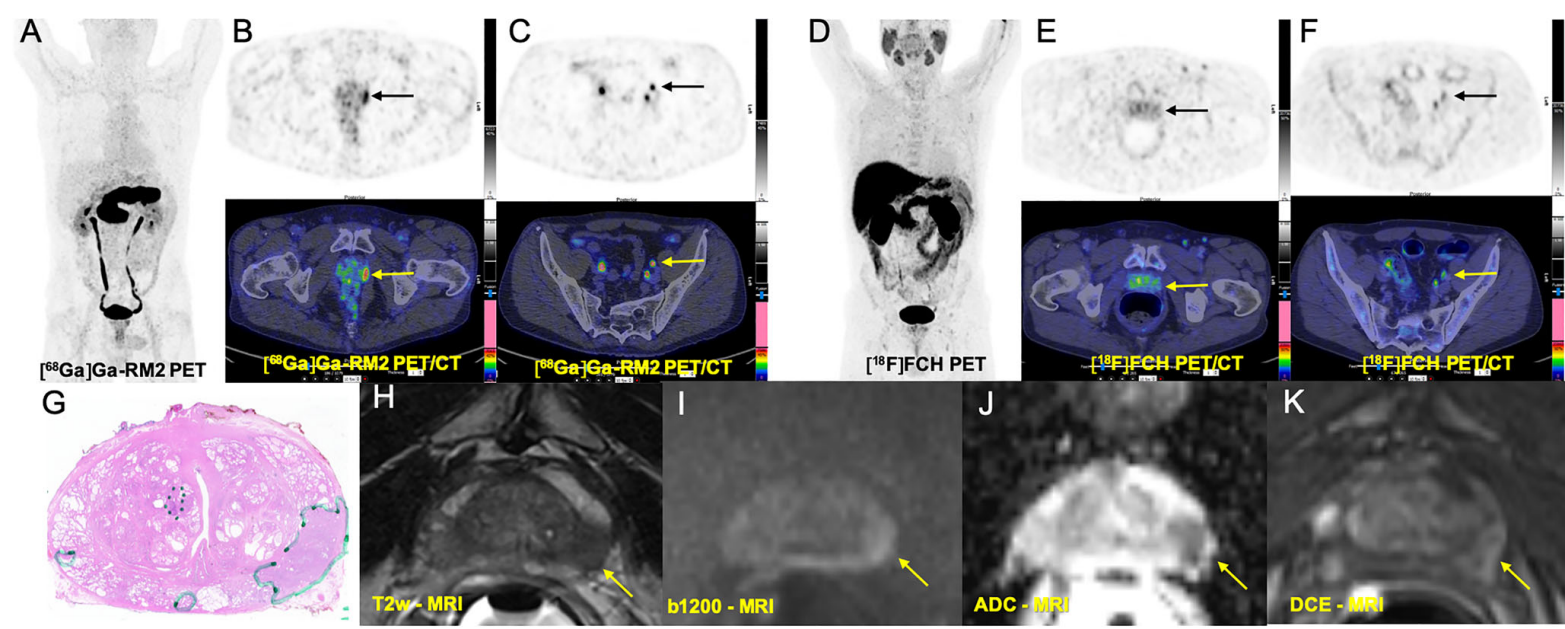
Comparison of [^68^Ga]Ga-RM2 PET-CT with [^18^F]FCH PET-CT and mpMRI in pre-operative staging of a prostate cancer patient with high risk of extraglandular metastases (PSA: 4.49 ng/ml, Gleason score: 9 (4 + 5), pT3a N1 (1/23) R0 L1 V0 Pn1). **A** [^68^Ga]Ga-RM2 PET: maximum intensity projection. **B** Axial view [^68^Ga]Ga-RM2 PET-CT (PET: upper, fusion PET-CT: lower) from the prostate region shows moderate focal tracer uptake on the left prostate lobe (arrows) corresponding to malignant finding on histopathology (**G**) (true positive). **C** Axial view [^68^Ga]Ga-RM2 PET-CT (PET: upper, fusion PET-CT: lower row) from the pelvis shows focal tracer uptake on a lymph node on the left iliac region (arrows). **D** [^18^F]FCH PET-CT MIP. **E** Axial view [^18^F]FCH PET-CT (PET: upper, fusion PET-CT: lower row) from the prostate region shows no remarkable tracer uptake on the prostate (arrows) (false negative). **F** Axial view [^18^F]FCH PET-CT from the pelvis shows only faint focal tracer uptake on the lymph node on the left iliac region (arrows). T2-weighted image (**H**) demonstrats a focal area of decreased signal in the left peripheral zone (yellow arrow) with corresponding diffusion-weighted imaging signal restriction (trace *b* values of 1200 s/mm^2^, **I**; ADC, **J**) and early contrast enhancement (dynamic contrast enhancement, DCE, **K**). Overall, these findings represent a PI-RADs version 2.1 score 5 lesion

**Fig. 5:**
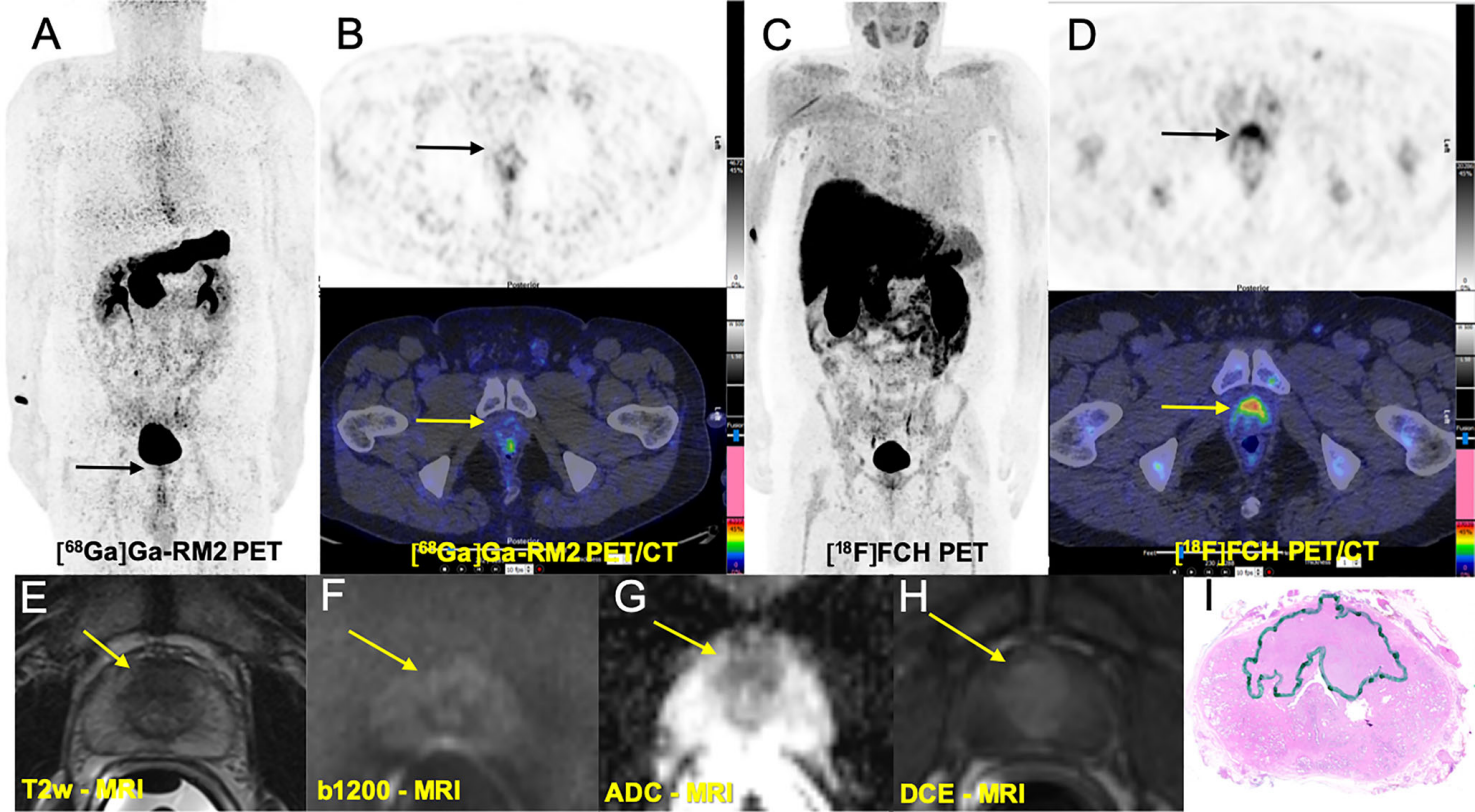
Comparison of [^68^Ga]Ga-RM2 PET-CT with [^18^F]FCH PET-CT and mpMRI in pre-operative staging of a prostate cancer patient with high risk of extraglandular metastases (PSA: 21.6 ng/ml, Gleason score: 7 (4 + 3), pT3a pN0 pR1 L0 V0 pn1. **A** [^68^Ga]Ga-RM2 PET: maximum intensity projection. **B** Axial view [^68^Ga]Ga-RM2 PET-CT (PET: upper, fusion PET-CT: lower row) from the prostate region shows no remarkable tracer uptake on the (arrows) (false negative). **D** [^18^F]FCH PET-CT MIP. **E** Axial view [^18^F]FCH PET-CT (PET: upper, fusion PET-CT: lower row) from the prostate region shows intensive tracer uptake at the apical anterior part of the prostate (arrows) corresponding to malignant finding on histopathology (**I**) (true positive). T2-weighted image (**E**) demonstrated a focal area of decreased signal in the central gland, anterior to the urethra (yellow arrow) with corresponding diffusion-weighted imaging signal restriction (trace *b* values of 1200 s/mm^2^, **F**; ADC, **G**) and early contrast enhancement (dynamic contrast enhancement, DCE, **H**). Overall, these findings represented a PI-RADs version 2.1 score 5 lesion

High physiological [^68^Ga]Ga-RM2 uptake was recorded in the pancreas and, because of its renal excretion, in the urinary tract. Mildly to moderately increased uptake was observed in the gastrointestinal tract. The liver, spleen, and bone marrow showed no noticeable physiological uptake (Figs. 2, 3, 4, and 5A).

### Primary tumor

[^68^Ga]Ga-RM2 PET-CT was able to detect at least 1 index lesion in the prostate gland of 83% (20/24) of patients with positive results in the final histopathology, while [^18^F]FCH PET-CT and mpMRI detected at least 1 index lesion in 76% (16/21) and 96% (23/24) of patients, respectively (Fig. 2).

In 2 low-risk patients with initially positive biopsy reports, final histopathology showed only 2- and 5-mm microfoci of Gleason 3 + 3 carcinoma but no clinical significance for the final correlation analyses with imaging findings.

### Region-based analysis

Of the overall 312 analyzed regions, 120 regions (4 to 8 lesions per patient) showed abnormal findings in the prostate gland either on imaging modalities and/or on histopathology. Histopathological findings confirmed cancerous tissue in 50 (42%) regions, while 70 regions showed no evidence of tumor infiltration (Table 1). Overall, sensitivity and specificity of [^68^Ga]Ga-RM2 PET-CT, [^18^F]FCH PET-CT, and mpMRI in the detection of primary tumor were 74% [95% confidence interval (95% CI): 62–86] and 90% (95% CI: 83–97), 60% (95% CI: 46–74) and 80% (95% CI: 70–90), and 72% (95% CI: 60–84) and 89% (95% CI: 81–96), respectively. The sensitivity and specificity of each imaging modality in low-, intermediate-, and high-risk patients are presented in Table 1. Although the overall sensitivity of [^68^Ga]Ga-RM2 PET-CT was higher compared to that of [^18^F]FCH PET-CT and mpMRI, the statistical analysis showed only significant difference between [^68^Ga]Ga-RM2 PET-CT and [^18^F]FCH PET-CT in the intermediate-risk group (*p* = 0.01) and [^68^Ga]Ga-RM2 PET-CT and mpMRT in the high-risk group (*p* = 0.03).

**Table 1:** Details of positive and negative histopathological findings in the region-based analysis and sensitivity and specificity of each imaging modality in low-, intermediate-, and high-risk patients

Region	Number/Percentage	Positive	Negative
Overall	**120**	**50 (42%)**	**70 (58%)**
Low risk	36	13 (36%)	23 (64%)
Intermediate risk	40	20 (50%)	20 (50%)
High risk	44	17 (39%)	27 (61%)
Sensitivity	**[**^**68**^**Ga]Ga-RM2**	**[**^**18**^**F]FCH**	**mpMRI**
Overall	**74%**	**60%**	**72%**
Low risk	77%	69%	69%
Intermediate risk	80%	42%	55%
High risk	65%	73%	94%
Specificity	**[**^**68**^**Ga]Ga-RM2**	**[**^**18**^**F]FCH**	**mpMRI**
Overall	**90%**	**80%**	**89%**
Low risk	83%	61%	87%
Intermediate risk	90%	94%	85%
High risk	96%	90%	93%

### Lesion-based analysis

Overall, 56 lesions were analyzed in prostate glands on [^68^Ga]Ga-RM2 PET-CT and mpMRI and 51 lesions on [^18^F]FCH PET-CT. The difference between analyzed lesions was due to lack of performance of [^18^F]FCH PET-CT in 3 patients. Histopathological results showed prostate cancer in 39 lesions. The sensitivity of [^68^Ga]Ga-RM2 PET-CT, [^18^F]FCH PET-CT, and mpMRI in the detection of primary tumor was 74% (95% CI 61–88), 61% (95% CI 45–77), and 67% (95% CI 52–82), respectively. The sensitivity and positive predictive value of each imaging modality in low-, intermediate-, and high-risk patients are displayed in Table 2.

**Table 2:** Lesion-based analysis: sensitivity, mean of maximum standardized uptake value (mean SUVmax), and positive predictive value (PPV) of each imaging modality in low-, intermediate-, and high-risk patients

Sensitivity	**[**^**68**^**Ga]Ga-RM2 (mean SUVmax,** ***p*** **value)**	**[**^**18**^**F]FCH (mean SUVmax)**	**mpMRI**
Overall	**74% (5.98 ± 4.13;** *p* ** = 0–13)**	**61% (6.08 ± 2.74)**	**67%**
Low risk	77% (6.78 ± 5.07; *p* = 0.68)	62% (6.05 ± 2.11)	62%
Intermediate risk	80% (6.16 ± 3.67; *p* = 0.13)	50% (5.16 ± 1.63)	53%
High risk	64% (4.66 ± 2.60; *p* = 0.19)	78% (6.45 ± 3.30)	91%
PPV	**[**^**68**^**Ga]Ga-RM2**	**[**^**18**^**F]FCH**	**mpMRI**
Overall	**81%**	**67%**	**79%**
Low risk	71%	50%	80%
Intermediate risk	80%	50%	53%
High risk	64%	78%	91%

Overall, there was no significant difference between the SUVmax of [^68^Ga]Ga-RM2 and [^18^F]FCH PET-CT in the intraprostatic malignant lesions ([^68^Ga]Ga-RM2: mean SUVmax 5.98 ± 4.13, median: 4.75; [^18^F]FCH: mean SUVmax 6.08 ± 2.74; median: 5.5; *p* = 0.13). However, the mean SUVmax of index lesions was significantly higher on [^68^Ga]Ga-RM2 compared to that of [^18^F]FCH PET-CT ([^68^Ga]Ga-RM2: mean SUVmax 7.89 ± 4.94; [^18^F]FCH: mean SUVmax 5.34 ± 2.52; *p* = 0.03). A differentiation between malignant and BPH was not possible using an SUV-cutoff neither on [^68^Ga]Ga-RM2 nor on [^18^F]FCH PET-CT. However, the tumor to background ratio was 2.5 on ^68^Ga-RM2 PET-CT compared to 2.0 on [^18^F]FCH PET-CT (*p* = 0.21). Although the mean SUVmax was higher in the low-risk and intermediate-risk compared to that in the high-risk patients on [^68^Ga]Ga-RM2 PET-CT, the difference was not statistically significant and we did not find any SUV cutoff in order to predict the risk classification (Table 2). Also, there was no significant difference between mean SUVmax of various risk groups on [^18^F]FCH PET-CT (Table 2).

### Lymph node and distant metastases

Lymph node metastases were detected in 2 patients. In 1 high-risk patient with a PSA value of 30 ng/ml and Gleason score of 8 (4 + 4) with 4 regional lymph node metastases, [^18^F]FCH PET-CT was able to detect 1 additional lymph node (diameter 15 mm) in the internal iliac region, which was negative on [^68^Ga]Ga-RM2 PET-CT. Overall, the detected lymph nodes on [^18^F]FCH PET-CT showed markedly higher uptake (mean SUVmax: 13.5 ± 3.72) compared to those detected on [^68^Ga]Ga-RM2 PET-CT (mean SUVmax: 3.92 ± 0.70) (Fig. 3). In contrast, in another high-risk patient with a PSA value of 4.9 ng/ml and Gleason score of 9 (4 + 5), [^68^Ga]Ga-RM2 PET-CT detected 1 metastatic regional lymph node with a diameter of 9 mm in the external iliac region (SUVmax: 6.9), which was negative on [^18^F]FCH PET-CT. No distant metastases were detected in our patient population (Fig. 4).

## Discussion

There are currently tremendous efforts on developing novel PET radiotracers, which can accurately detect intraprostatic cancer and differentiate malignant from BPH and inflammatory lesions.

The aim of the present study was to investigate the diagnostic accuracy of [^68^Ga]Ga-RM2 PET-CT compared to that of ^18^F-FCH PET-CT and mpMRI in the detection of primary prostate cancer. Furthermore, the pattern of tracer uptake on PET-CT was correlated with tumor characteristics on histopathology in low-, intermediate-, and high-risk PCa patients.

In a region-based analysis, the sensitivity and specificity of [^68^Ga]Ga-RM2 PET-CT were superior to those of ^18^F-FCH PET-CT and comparable to those of mpMRI. In overall assessment, there was no significant difference in the diagnostic accuracy of the different modalities. However, [^68^Ga]Ga-RM2 PET-CT showed significantly higher sensitivity compared to [^18^F]FCH PET-CT in intermediate-risk prostate cancer patients and mpMRI revealed significantly higher sensitivity in high-risk cases. In the lesion-based analysis, overall, 39 PCa lesions were defined in histopathology. [^68^Ga]Ga-RM2 PET-CT showed superior sensitivity of 74% in the detection of primary tumor compared to [^18^F]FCH PET-CT and mpMRI with a sensitivity of 61% and 67%, respectively. The overall findings of the present investigation are in concordance with those of similar studies [10, 18, 20, 23, 24]. To our best knowledge, this is the first prospective clinical investigation that explicitly evaluates the impact of [^68^Ga]Ga-RM2 PET-CT and compare its value with that of [^18^F]FCH PET-CT in three patients’ cohorts with different metastatic risk stratifications, in which all patients underwent radical prostatectomy. When correlating the diagnostic accuracy of [^68^Ga]Ga-RM2 PET-CT with biological tumor characteristics and metastatic risks, we noticed, both on region- and lesion-based analyses, limited sensitivity of 65% in the high-risk patient’s group compared to 74% for [^18^F]FCH PET-CT and 94% for mpMRI (Fig. 5). In contrast, the [^68^Ga]Ga-RM2 PET-CT showed significantly higher sensitivity of 80% in intermediate-risk cases compared to 42% and 55% for [^18^F]FCH PET-CT and mpMRI, respectively. These findings are not in line with the data presented by Kähkönen et al, who reported markedly higher sensitivity of [^68^Ga]Ga-RM2 PET-CT of 88% for the detection of primary tumor in 11 high-risk PCa patients [18]. This different sensitivity might be explained by the more advanced tumor stage in that study, as higher T-categories were reported in their patients compared to our high-risk cohort [23]. Nevertheless, an accurate conclusion cannot be drawn because of the low number of the patients in both studies.

In the last years, tremendous investigations have been done for developing radioligands targeting prostate-specific-membrane antigen (PSMA) for the depiction of malignant tissues particularly in prostate cancer and several small-molecule tracers targeting PSMA have generated a lot of interest [25–27].

[^68^Ga]Ga-PSMA and [^18^F]PSMA tracers in conjunction with both PET-CT and PET/MRI have emerged as promising imaging modalities for primary staging and restaging of PCa [28–31]. However, there are still limited prospective data on the impact of ^68^Ga-labeled PSMA PET-CT for intraglandular detection of PCa [32]. A subanalysis of prospective data of [^68^Ga]Ga-PSMA PET/MRI in PCa showed significant differences in tracer uptake of the dominant intraprostatic cancer tissue between postoperative low/intermediate-risk patients and high-risk subjects [31]. In a recent preclinical study, the authors retrospectively compared the binding of radiolabeled GRPr-antagonists (i.e., [^111^In]RM2) with [^111^In]PSMA-617 in 20 frozen prostatectomy samples with various metastatic risks of the D’Amico classification [33]. They reported a significantly higher binding affinity of [^111^In]RM2 in low-metastatic-risk samples with low Gleason score and low PSA value, while the binding of [^111^In]PSMA-617 was high in almost all cancerous tissues independent of metastatic risk, Gleason score, or PSA value. The authors concluded that GRPr and PSMA-based imaging may have a complimentary role to fully characterize prostate cancer disease, GRPr being targeted in low-metastatic-risk patients while PSMA could be a valuable target in higher risks.

The findings of the current study support the data from previous investigations showing that [^68^Ga]Ga-RM2 PET-CT detected more intraglandular prostate cancer lesions in the low-risk group. However, we found no correlation between tracer uptake by means of SUVmax on [^68^Ga]Ga-RM2 and [^18^F]FCH PET-CT and Gleason score, PSA value, and risk of metastases, in line with previous clinical reports [20, 23]. There was also no relevant trend in the increasing pattern of SUVmax relating to PSA value and/or Gleason score. Although the results of latter preclinical investigation agree with the known increased GRPr expression in low-grade prostate cancer, in vitro and preclinical results may not necessarily represent the imaging findings on humans.

In one of the early studies evaluating the impact of ^68^Ga-labeled Bombesin antagonists in prostate cancer, the authors observed a significant difference in SUV between cancerous and hyperplastic prostatic lesions [18]. Although less false-positive intraprostatic lesions were seen on [^68^Ga]Ga-RM2 PET compared to [^18^F]FCH PET (i.e., 7 versus 11, respectively), a differentiation between malignant and BPH was not possible using a SUV-cutoff neither by [^68^Ga]Ga-RM2 nor [^18^F]FCH PET-CT. The different findings may be related to the selection bias, as most of the patients undergoing surgery in that study belonged to the high-clinical-risk group with high risk of lymph node metastasis.

Touijer et al recently published clinical data in 16 patients with biopsy-proven primary PCa with low (*n* = 2), intermediate (*n* = 8), and high risk (*n* = 6) of recurrence. The sensitivity, specificity, and accuracy of 85.2%, 81.3%, and 83.9% were reported for fused [^68^Ga]Ga-RM2 PET-CT-mpMRI. The average SUVmax ranged from 1.5 to 27.8 (mean: 9.1) for dominant tumors and 0.45 to 7.1 (mean: 3.7) for BPH. However, no correlation was found between SUVmax and Gleason score [20]. The higher diagnostic performance can be explained by using both [^68^Ga]Ga-RM2 PET-CT and mpMRI findings and the higher number of patients with intermediate risk.

Despite the intention of gaining information on extraprostatic metastases in the high-risk group, only two cases out of the 10 showed metastases, so limited conclusions can be made. In the detection of lymph node metastases, [^68^Ga]Ga-RM2 and [^18^F]FCH PET-CT showed contradicting findings in two high-risk PCa patients (Figs. 3 and 4). This may be related to the PSA value of these patients; however, because of the limited number of patients with lymph node metastases, the impact of [^68^Ga]Ga-RM2 PET-CT in the assessment of lymph node metastases remains inconclusive.

Our findings in this study may have future clinical impact. Prostate cancer patients with low metastatic risk are today not eligible for radical treatments anymore but rather to active surveillance or local treatments [34]. In addition, Gleason score is upgraded in about 30% of PCa patients between biopsies and radical prostatectomy [35]. Thus, an imaging procedure capable to discriminate “true” low and intermediate from high metastatic risks would be helpful to schedule local treatments in this group of patients. Results of this work indicate that GRPr targeting by hybrid imaging (e.g. PET/MRI) seems promising procedure amenable to better biopsy guidance in low- and intermediate-metastatic-risk PCa patients and has the potential to discriminate them from PCa patients with higher risks. In addition, GRPr-based imaging seems to play a complementary role to PSMA-based or Choline-based imaging for fully characterization of prostate cancer disease.

The main limitation of this study was the small number of patients in each risk group, mainly due to the exploratory design of this research. Thus, given the exploratory nature of this phase I/II study and the relatively small sample size, *p* values without correction for multiple comparisons were reported as guidance, but they should not be interpreted as a formal hypotheses testing. In addition, [^18^F]FCH appears to be outperformed by PSMA-radiotracers in light of evolving data showing the value of PSMA-based PET-CT in PCa. This could moderate the clinical impact of this study.

## Conclusion

[^68^Ga]Ga-RM2 is a promising new PET tracer with a high detection rate for intraprostatic PCa. In addition, GRPr-based imaging can play a complementary role to choline-based or PSMA-based imaging for full characterization of prostate cancer disease and biopsy guidance in low- and intermediate-metastatic-risk PCa patients and has the potential to discriminate them from those of higher risks.

## Supplementary Information

ESM 1 (DOCX 18 kb)

ESM 2 (DOCX 37 kb)

## Abbreviations

[^18^F]FCH: [^18^F]fluoromethylcholine
[^68^Ga]Ga-RM2: Gallium-68 - DOTA-4-amino-1-carboxymethylpiperidine-D-Phe-Gln-Trp-Ala-Val-Gly-His-Sta-Leu-NH2
[^18^F]DCFPyl: 2-(3-{1-Carboxy-5-[(6-[^18^F]fluoro-pyridine-3-carbonyl)-amino]-pentyl}-ureido)-pentanedioic acid
[^68^Ga]Ga-HBED-PSMA: ^68^Ga-Glu-NH-CO-NH-Lys(Ahx)-HBED-CC
^11^C: Carbon-11
^18^F: Fluorine-18
ADC: Apparent diffusion coefficient
BPH: Benign prostatic hyperplasia
CI: Confidence interval
CT: Computed tomography
DCE: Dynamic contrast enhanced
DWI: Diffuse weighted image
GRP: Gastrin-releasing-peptide receptor
LN: Lymph node
MIP: Maximum intensity projection
mpMRI: Multiparameteric prostate magnetic resonance imaging
NCCN®: National Comprehensive Cancer Network®
PCa: Prostate carcinoma
PET: Positron emission tomography
PI-RADS: Prostate Imaging–Reporting and Data System
PSA: Prostate-specific antigen
PSMA: Prostate-specific membrane antigen
RM2: DOTA-4-amino-1-carboxymethylpiperidine-D-Phe-Gln-Trp-Ala-Val-Gly-His-Sta-Leu-NH2
SPSS: Statistical Package for the Social Sciences
SUVmax: Maximum standardized uptake value
T1W: T1-weighted
T2W: T2-weighted
TRUS: Transrectal ultrasound
TSE: Turbo spin echo

## Declarations

### Guarantor

The scientific guarantor of this study is Mohsen Beheshti MD, FEBNM, FASNC, professor and head of the Division of Molecular Imaging and Theranostics, Department of Nuclear Medicine & Endocrinology, University Hospital Salzburg, Paracelsus Medical University, Salzburg, Austria

### Statistics and biometry

One of the authors has significant statistical expertise.

### Methodology

• Prospective

• Diagnostic or prognostic study

• Multicenter study
