# Supplementary material for: Value of 68Ga-labeled bombesin antagonist (RM2) in the detection of primary prostate cancer comparing with [18F]fluoromethylcholine PET-CT and multiparametric MRI—a phase I/II study
Source: Eur Radiol. 2022 Jul 21;33(1):472–82. doi: 10.1007/s00330-022-08982-2 (PMC9755087; doi:10.1007/s00330-022-08982-2)
Supplement: Supplementary file 1 — (DOCX 18 kb) [file 330_2022_8982_MOESM1_ESM.docx]

**Supplement 1:** Inclusion and exclusion criteria for selecting the patients.

| **Inclusion criteria** | **Exclusion criteria** |
| --- | --- |
| 1. Written informed consent.  2. Males ≥ 45 years of age.  3. Patients with diagnosis of primary prostate cancer in which prostate cancer is histologically confirmed and results of histology are available.  4. Patient with planned prostatectomy (within 4 weeks following the [68Ga]RM2 scan).  5. Patient had a MRI, and [18F]-choline PET/CT (when available), for primary detection or staging and the images and the results are available (Note: [18F]-choline PET/CT is optional).  6. The MRI and [18F]-choline PET/CT referred to in criterion 5 were performed preferably within not more than 5 days prior to the planned imaging with [68Ga]RM2. The maximum interval between MRI and [18F]-choline PET/CT and treatment with [68Ga]RM2 PET/CT is 6 weeks.  7. No chemotherapy, radiotherapy, biopsy or immune/biologic therapy between MRI and [18F]-choline PET/CT (when performed) and [68Ga]RM2 PET/CT performed or scheduled.  8. Recovery (excluding alopecia) from previous surgery, radiation, and chemotherapy  9. ECOG (Eastern Cooperative Oncology Group) performance status of <1  10. No clinically relevant deviations in renal function as determined by Cockcroft and Gault method using serum creatinine at screening.  11. No malfunction equivalent to CTC (Common toxicity criteria) toxicities grade > 2 of the liver (ALT; bilirubin).  12. Life expectancy of at least 3 months. | 1. Previous transurethral resection of the prostate (TUR-P)  2. Concurrent severe and/or uncontrolled and/or unstable medical disease other than prostate cancer (e.g. poorly controlled diabetes, congestive heart failure, myocardial infarction within 12 months prior to planned injection of [68Ga]RM2, unstable and uncontrolled hypertension, chronic renal or hepatic disease, severe pulmonary disease) which could compromise participation in the study.  3. Known sensitivity to the study drug or components of the preparation.  4. Patient is in custody by order of an authority or a court of law.  5. Patient is a relative of the investigator, student of the investigator or otherwise dependent.  6. Patient is participating in another clinical study involving administration of an investigational drug at the same time as well as in the preceding 4 weeks before radiotracer administration. Participation in another clinical study involving administration of an investigational drug has ended within the preceding 4 weeks  before radiotracer administration.  7. Unwillingness or inability to comply with the protocol.  8. Patient fulfils criteria which in the opinion of the investigator preclude participation for scientific reasons, for reasons of compliance, or for reasons of the patient’s safety.  9. Hematological or biochemical parameters that are outside the normal range and are considered clinically significant by the investigator, i.e. CTC (Common toxicity criteria) toxicities grade > 2. Minor deviations in lab parameters that are considered by the evaluating physician to be not clinically significant with respect to safety or interpretation of study results are not considered an exclusion criterion.  10. History of significant occupational exposure to ionizing radiation or monitoring of occupational radiation exposure (according to recommendations from current guidelines).  11. Donation of blood within 12 weeks or plasmapheresis within 2 weeks before the radiotracer administration. |
