# Supplementary material for: Value of 68Ga-labeled bombesin antagonist (RM2) in the detection of primary prostate cancer comparing with [18F]fluoromethylcholine PET-CT and multiparametric MRI—a phase I/II study
Source: Eur Radiol. 2022 Jul 21;33(1):472–82. doi: 10.1007/s00330-022-08982-2 (PMC9755087; doi:10.1007/s00330-022-08982-2)
Supplement: Supplementary file 2 — (DOCX 37 kb) [file 330_2022_8982_MOESM2_ESM.docx]

**Supplement 2**

***PET-CT Imaging***

The study was performed with dedicated PET-CT scanners (GE Healthcare, 23 patients: Discovery 710; 3 patients: Discovery VCT). All patients fasted for at least 4 hours before each examination. Preparation of study subjects consisted of inserting a suitable indwelling intravenous catheter into a large vein (e.g., antecubital vein), preferably in the subject’s nondominant arm. Correct localization of the indwelling cannula must be ensured by test injection of normal saline solution prior to injecting the study drug.

After injecting the study drug, the cannula and injection system were flushed with 10 ml saline solution. Imaging was acquired 60 min. after an intravenous (i.v.) injection of 4 mCi (148 MBq) of ^68^Ga-RM2. All except 3 patients, had concomitant ^18^F-FCH PET-CT imaging 60 min. after i.v. administration of 6 mCi (222 MBq) ^18^F-FCH. All patients received standard volume (i.e. 250 ml) of sodium chloride i.v. infusion and 250-500 ml oral fluid before the PET-CT acquisition in order to push tracer excretion for better image quality and reduce the radiation exposure in the urinary system.

PET acquisitions were obtained from the base of the skull to the proximal of the thigh with 2.5 min./bed position acquisition time using time of flight (TOF) modus. All images were reconstructed identically with ordered-subsets expectation maximization algorithm (4 iterations, 18 subsets) followed by a post-reconstruction smoothing Gaussian filter (4.0 mm in full width at one-half maximum).

The CT portion of the ^18^F-FCH PET-CT procedure was performed after intravenous infusion of 100 ml ionic contrast medium with high beam current modulation (120-330 mA, 0.6 second per rotation, 5.0 mm reconstructed section thickness, 0.5 mm overlap, 512x512 matrix, pitch index 1.5), while a non-contrast CT with low beam current modulation (80–120 mA) was obtained on 68Ga-RM2 PET-CT for localization and attenuation correction. The reformatted, transverse, coronal, and sagittal views were used for interpretation.

Images were read using advanced PET-CT review software (Advantage Windows, version 4.6; GE Medical Systems), which allows simultaneous scrolling through the corresponding PET, CT, and fusion images in the transverse, coronal, and sagittal planes. The prostate gland contour was identified on the PET images based on corresponding CT images. The defined prostate volume on PET images was extracted using a software program provided by the manufacturer.

One experienced nuclear medicine specialist from each center, who only had knowledge of each patient’s diagnosis interpreted all of the PET scans. Further, they were given access to the CT as well as PET-CT fusion images for morphological correlation and localization of pathological PET lesions. All discordant findings have been evaluated in a multidisciplinary consensus meeting with related specialists of both centers for final interpretation.

Prostate gland was classified in 12 anatomic segments (apical, middle, and basal thirds, each divided into four segments of right, left, anterior, and posterior), for data analysis of both imaging as well as histopathologic findings.

A lesion was considered pathologic when the focal tracer accumulation was greater than the background activity. Semi-quantitative analysis of the abnormal radiotracer uptake was performed using the maximum standardized uptake value (SUVmax) within the volume of interest, manually placed over the pathological lesions on each anatomic section. The segment with the highest tracer intensity was defined as “index lesion” for the correlation with the segment with maximum tumor involvement on histopathology.

**Supporting Matherial MRI data acquisition**

The MRI protocol at each institution consisted of T1W turbo spin echo (TSE) sequence in axial plane to exclude post-bioptic hemorrhage, T2W TSE sequences in axial (orthogonal to the urethra), coronal and sagittal planes. Diffuse weighted image (DWI) performed with echo-planar read-out obtained in transverse plane parallel to the transverse T2W to with apparent diffusion coefficient (ADC) maps reconstructed using the standard manufacture software. The 3D volume of the entire prostate was covered.

Dynamic contrast enhancement 3D T1-spoiled gradient echo (GE) images were acquired during an intravenous bolus injection of paramagnetic contrast medium at a dose of 0.2 mmol/kg of body weight for examination at 1.5T and 0.1 mmol/kg of body weight for examination at 3T. A common flow rate of 2.0 ml/s was used, followed by a 20 ml saline flush. The images were acquired every 13 seconds at 1.5T and every 7 seconds at 3T, with each sequence lasting for 4:30 and 6:59 minutes at 1.5T and 3T, respectively.

In order to suppress bowel peristalsis, 1.0 mg of glucagon (GlucaGen, Novo Nordisk, Bagsværd, Denmark) subcutaneously was given immediately before the start of the examination. Moreover, patients were instructed to take laxatives in the evening before the mpMRI examination to limit rectal air (Laxoberon [Boehringer Ingelheim, Ingelheim, Germany]) as a part of routine preparation for prostate MRI protocol.

Anatomical triplanar T2-weighted images were acquired using a turbo spin-echo sequence with repetition time/echo time (TR/TE) 6400-8640/101 ms, field of view (FOV) 200 x 200 mm^2^, matrix size 320 x 320, slice thickness 3 mm, no gaps, number of signal averages (NSA) 2 and acquisition time 2.27 min (transverse images), 2.47 min (sagittal images), 2.78 min (coronal images). Generalized autocalibrating partially parallel acquisitions (GRAPPA) parallel imaging method[1] was used with the acceleration factor of 2 and 32 reference lines for autocalibration. A single shot spin-echo based sequence was used for DWI with TR/TE 5543/80 ms, FOV 260 x 260 mm^2^, matrix size 128 x 128, slice thickness 3 mm, no gaps NSA 4, a bandwidth 1184Hz/pixel acquiring 6/8 (75%) of k-space in phase-encoding direction, b-values 0, 100, 200 350, 500 s/mm², diffusion gradients in three orthogonal directions for each b-value, GRAPPA with the acceleration factor of 2 and 32 reference lines. The acquisition time for DWI was 5.1 min. Additional DWI with 16 b values (0, 50, 100, 200, 350, 500, 650, 800, 950, 1100, 1250, 1400, 1550, 1700, 1850, 2000 s/mm²) was acquired with TR/TE 7000/87, FOV 200 x 200 mm2, matrix size 320 x 320, slice thickness 5 mm, NSA 2 and acquisition time 11.07 min. The apparent diffusion coefficient maps were calculated automatically by vendor specific software using mono-exponential fit with a noise level of 10,. The three-dimensional ^1^H-MRS covering whole prostate was performed using point resolved spatially localized spectroscopy (PRESS) sequence. Automatic and manual shimming of whole prostate volume was performed (to optimize the main magnetic field homogeneity) in every patient. Weighted averaging (NSA: 6) of elliptically sampled k-space, Hanning filtering of the signal and zero-filling to a 16 x 16 matrix was performed before Fourier transformation[2]. The TR and TE were optimized for the shape of citrate resonance (3,4). Additional water and lipid signal from adjacent tissues was suppressed with seven outer volume saturation slabs. The following additional imaging parameters were used: acquisition bandwidth 1300 Hz, 512 spectra data points, FOV 96 x 96 x 96 mm^2^ and matrix size of 12 x 12 x 12 resulting into nominal voxel size of 8x8x8 mm^3^. A real voxel size could be best approximated as a sphere with a volume of 1.51 cm^3^ and diameter of 14.24 mm after apodization [3]. The acquisition time was about 15-20 minutes including manual shimming. Axial dynamic contrast enhanced images were acquired before, during and after injection of contrast agent. Contrast agent (0.1 mmol/kg Dotarem [Guerbet, France] was injected 30 s after the beginning of the sequence through a peripheral vein at a rate of 2 ml/s via a mechanical injector (Spectris, Medrad, Indianola, USA). In total, 60 time points at a temporal resolution of 6.9 seconds were acquired using a three-dimensional VIBE sequence [4] with the following parameters: TR/TE 5.43/1.87 seconds, 15 degree flip angle, FOV 240 x 240 mm^2^, matrix size 192 x 192, slice thickness 3 mm, a bandwidth of 260Hz/pixel acquiring 6/8 (75%) of k-space in phase-encoding direction, GRAPPA with the acceleration factor of 2 and 24 reference lines, acquisition time 6.9 min. Before the contrast enhanced MR imaging, images with five different flip angles of 2, 5, 8, 10, 15 degrees were obtained for calculation of pre-contrast longitudinal relaxation time (T_10_) . The total duration of the MRI examination was about 60 minutes.

The radiologists had knowledge of the confirmation of the tumour by way of biopsy; no information regarding the clinical stage and prostate specific antigen level was available. T2W and DWI images including ADC maps, and DCE results were interpreted qualitatively based on PI-RADS (version 2.1) scoring system for the tumor detection and localization.

### **Histopathological Analysis**

Prostatectomy specimens were fixed in 10% buffered formalin for 24–48 hours and surgical margins were marked with different colors of tissue-ink to preserve the orientation and to allow for correlation with imaging datasets. Whole‐mount prostatectomy macro‐sections were obtained at 4–6‐mm intervals transversely in a plane perpendicular to the long axis of the prostate gland in the superior–inferior direction. The most apical and basal macro‐sections were further sectioned in coronal orientation to evaluate any extraprostatic cancer extension at apex/basis or seminal vesicle invasion. Four μm sections from each paraffin block were cut, stained with hematoxylin–eosin and reviewed by two board‐certified genitourinary pathologists. Gleason scores were assigned to all the lesions as combinations of primary, secondary, and tertiary Gleason grade, as defined by the 2014 International Society of Urological Pathology Modified Gleason Grading System [5]. If a Gleason grade pattern higher than the primary and secondary grade was present and visually accounted for less than 5% of the tumor volume, it was assigned as tertiary Gleason grade. Only PCa lesions with a diameter > 5 mm were considered clinically significant in the final correlation analyses with imaging findings.

**References**

1 Griswold MA, Jakob PM, Heidemann RM et al (2002) Generalized autocalibrating partially parallel acquisitions (GRAPPA). Magn Reson Med 47:1202-1210

2 Scheenen TW, Klomp DW, Roll SA, Futterer JJ, Barentsz JO, Heerschap A (2004) Fast acquisition-weighted three-dimensional proton MR spectroscopic imaging of the human prostate. Magn Reson Med 52:80-88

3 Scheenen TW, Heijmink SW, Roell SA et al (2007) Three-dimensional proton MR spectroscopy of human prostate at 3 T without endorectal coil: feasibility. Radiology 245:507-516

4 Scheenen TW, Gambarota G, Weiland E et al (2005) Optimal timing for in vivo 1H-MR spectroscopic imaging of the human prostate at 3T. Magn Reson Med 53:1268-1274

5 Epstein JI, Amin MB, Reuter VE, Humphrey PA (2017) Contemporary Gleason Grading of Prostatic Carcinoma: An Update With Discussion on Practical Issues to Implement the 2014 International Society of Urological Pathology (ISUP) Consensus Conference on Gleason Grading of Prostatic Carcinoma. Am J Surg Pathol 41:e1-e7
